# Thermoregulation in hot-dry and warm-humid heat stress during cycling among males

**DOI:** 10.3389/fphys.2026.1856143

**Published:** 2026-08-05

**Authors:** Xiujing Zhao, Brendon P. McDermott, Cory L. Butts

**Affiliations:** 1 Heat & Hydration Optimization (H2O) Lab^2^, University of Arkansas, Fayetteville, AR, United States; 2 Exercise Science Research Center, University of Arkansas, Fayetteville, AR, United States; 3 Department of Health, Human Performance & Recreation, University of Arkansas, Fayetteville, AR, United States

**Keywords:** biophysical, cardiovascular response, exercise, heat balance, thermal strain

## Abstract

**Introduction:**

Hot-dry (HD) and warm-humid (WH) environments with equivalent wet-bulb globe temperature (WBGT) may be expected to produce similar physiological strain; however, previous studies have reported divergent thermoregulatory and cardiovascular responses. This study compared heat storage, thermal, and cardiovascular responses between HD and WH conditions with matched WBGT during cycling.

**Methods:**

Ten healthy, active, non-heat-acclimatized males completed two randomized crossover trials consisting of 60 minutes of cycling at an individualized workload corresponding to 55% of peak power output in HD (39.2°C, 30% relative humidity [RH]) and WH (34.2°C, 55% RH) conditions with equivalent WBGT (~30°C). Heat storage, dry heat loss (DHL), and evaporative heat loss (EHL) were estimated using partitional calorimetry. Rectal temperature (T_re_), mean skin temperature (T_sk_), and cardiovascular responses were measured throughout the trials.

**Results:**

No condition differences were observed for cumulative heat storage, Tre, heart rate, blood pressure, or forearm skin blood flux (all p > .050). Tsk (HD: 36.28 ± 0.67°C, WH: 35.89 ± 0.70°C; p = .030, ηp² = .465) and EHL (HD: 450 ± 53 W, WH: 319 ± 30 W; p < .001, ηp² = .932) were greater in HD, whereas DHL was greater in WH (HD: −80 ± 25 W; WH: 48 ± 25 W; p < .001, ηp² = .950). Conclusion: Despite similar heat storage and cardiovascular strain, matched-WBGT environments altered the mechanisms of heat exchange. Thus, ambient temperature, humidity, and workload should be considered alongside WBGT when monitoring heat strain and selecting heat-mitigation strategies.

**Conclusion:**

Despite similar heat storage and cardiovascular strain, matched-WBGT environments altered the mechanisms of heat exchange. Thus, ambient temperature, humidity, and workload should be considered alongside WBGT when monitoring heat strain and selecting heat-mitigation strategies.

## Introduction

Global heatwaves, which have increased in frequency, duration, and intensity, have led to numerous health consequences over the last several decades (Perkins-Kirkpatrick and Lewis, 2020). An estimate suggests that around a third of globe’s population is exposed to deadly heatwaves and this percentage will increase to half by 2100 (Mora et al., 2017). With a warming climate, the potential for more frequent and severe heat events could significantly worsen heat-related morbidity and mortality (Cottle et al., 2022), especially for settings such as athletic (Gibson et al., 2020), occupational (Gubernot et al., 2014), and military operations (Moran et al., 2023) that occur during extreme heat stress with moderate to vigorous intensity physical activity. Notably, researchers found that heat disproportionately kills young people aged 18 to 34 years old (Ebi et al., 2021). Even though young adults are usually more physiologically robust to heat, social, economic, and behavioral factors can lead to higher heat-related mortality in this age group (Ebi et al., 2021).

Heatwaves have constantly threatened human health and safety (Gubernot et al., 2014). For instance, Europe sustained a severe heatwave that resulted in over 70,000 heat-related deaths in 2003 (Robine et al., 2008). Two notable heatwave types, namely, hot-dry (HD) and warm-humid (WH), endanger occupational activity, military operations, and athletic events. It is unclear, however, whether there is a difference in physiological stress and subsequent risk of heat-related illness between HD and WH heatwaves (Foster et al., 2022). It is imperative to differentiate the impact on physiological responses for these differing types of heat stress. Especially when a single metric (e.g., wet-bulb globe temperature [WBGT]) is used to quantify heat stress, regardless of type. In addition, a previous study reported that WBGT underestimates heat strain in high relative humidity (RH) environments (Budd, 2008). Others revealed that HD and WH conditions with matched WBGT induced divergent thermal and cardiovascular responses (Smolander et al., 1987; Keatisuwan et al., 1996; Seo et al., 2019; Bartman et al., 2024). It is necessary to identify heat strain between HD and WH conditions with identical heat stress values and subsequently help develop a targeted policy and safety strategy to mitigate future heat-related morbidity and mortality.

Furthermore, many previous studies have investigated the effect of heat stress on thermoregulatory and cardiovascular responses, mostly focused on minimal to low intensity physical activity due to the safety considerations (Kamon and Avellini, 1976; Kamon et al., 1978; Smolander et al., 1987; Kenney and Zeman, 2002; Wolf et al., 2021; Cottle et al., 2022; Foster et al., 2022). Cottle et al. (2022) found heat storage and gastrointestinal temperature were no different between HD and WH conditions for minimal and low intensity physical activity. Smolander et al. (1987) also indicated no differences in rectal temperature and heart rate between HD and WH conditions during low intensity walking. In contrast, Foster et al. (2022) suggested that physiological stress (heart rate, core temperature, and whole body sweat rate) was greater in HD conditions than in WH conditions during low intensity cycling. In consideration of the demand on adjusted safety requirements at varying exercise intensity, it is urgent to evaluate thermoregulatory and cardiovascular differences during more intense exercise with extreme heat stress.

Despite the general population reducing outdoor physical activity to avoid extreme heat stress, occupational groups (e.g., construction and utility workers), athletic events, and military operations occur in thermally stressful environments with moderate or vigorous activity. However, most studies comparing matched WBGT during exercise in the heat focus on minimal to low intensity (Smolander et al., 1987; Keatisuwan et al., 1996; Seo et al., 2019; Foster et al., 2022; Bartman et al., 2024). Given that it is inherent that moderate to vigorous activity occurs daily through recreation, industry and the military, it is imperative we investigate this work rate in terms of varying heat stress. This area of research is essential to develop, or confirm, safety for individuals in hot environments.

Therefore, the primary aim of this investigation was to compare thermoregulatory and cardiovascular responses between HD and WH conditions with matched WBGT during 60 minutes of moderate intensity cycling among healthy male adults. We hypothesized that heat storage, rectal temperature, and heart rate would not differ between HD and WH conditions. Still, there would be more dry heat gain and evaporative heat loss (EHL) in HD compared to WH conditions. Further, we assumed dry heat loss (DHL) would be greater in WH than in HD conditions.

## Materials and methods

### Participants

Fifteen healthy, active [physically active at least two days/week; peak oxygen consumption (VO_2peak_) ≥ 45 ml/kg/min] and non-heat-acclimatized males volunteered and completed this study. Data from five participants were excluded prior to analysis because of factors that could substantially influence physiological responses to exercise in the heat. Three participants subsequently disclosed illness that occurred during the experimental period after atypical physiological responses prompted additional questioning, and two participants did not comply with pre-trial preparation instructions (one did not refrain from exercise within 24 h of testing and one reported altered sleep prior to a trial). These exclusions were made to preserve participant safety and data integrity and were not based on the study outcomes. Demographics for our final N = 10 participants appear in **Table 1**.

**Table 1:** Participant characteristics (n = 10).

Characteristic	Mean (SD)	Range
Age (y)	29 (7)	19–39
Height (m)	1.79 (0.11)	1.62–1.92
Body Mass (kg)	77.4 (9.3)	59.9–87.3
Body Fat (%)	18.2 (5.6)	7.5–24.3
BMI (kg/m^2^)	23.9 (1.4)	21.2–25.7
A_D_ (m^2^)	1.96 (0.18)	1.64–2.15
A_D_/wt (m^2^/kg)	0.025 (0.001)	0.025–0.027
VO_2peak_ (mL/kg/min)	51.9 (5.3)	44.9–62.0
VO_2peak_ (L/min)	4.00 (0.53)	3.14–4.79

BMI, body mass index; A_D_, body surface area; A_D_/wt, body surface area-to-mass-ratio; VO_2peak_, peak oxygen consumption.

All experimental procedures were approved by the University Institutional Review Board prior to data collection (IRB approved protocol number: 2309490663). Written informed consent was obtained from all participants before participation. All participants completed a medical history questionnaire and verified no exclusion criteria. Participants were originally excluded if they self-reported previous heat illness within the past three years, had a current musculoskeletal injury, or were taking medications known to affect thermoregulation. We excluded participants who use tobacco or electronic cigarettes. Participants were not allowed to participate if they reported cardiovascular, metabolic, renal, gastrointestinal or liver disease on screening medical history forms. Only male participants were included because fluctuations in ovarian hormones across the menstrual cycle can influence thermoregulatory responses, including sweating, skin blood flux, and core temperature regulation (Charkoudian and Stachenfeld, 2016). Restricting the sample to males reduced physiological variability and allowed for a more controlled examination of environmental effects on thermoregulatory and cardiovascular responses. All participants were asked to refrain from alcohol for 24 hours leading into trials and caffeine ingestion was matched between trials. Strenuous activity was restricted for 24 hours before both preliminary and experimental trials. Participants wore a T-shirt or cycling jersey, shorts, socks, and cycling shoes that were matched for both trials.

### Preliminary trial

During a preliminary visit, participants were familiarized with the experimental protocol and a body composition was assessed via dual energy x-ray absorptiometry prior to the experimental trial (Lunar Prodigy, General Electric, Madison, WI, USA). VO_2peak_ was assessed via a cycle ergometer (Racer Mate, Seattle, WA) graded exercise protocol. Participants warmed up at 100W for five minutes at a self-selected cadence. Following the warm-up, resistance was increased by 25-50W every two minutes until the participant could not maintain 60 revolutions per minute (RPM) on the cycle ergometer. During the VO_2peak_ test, heart rate (Tango II, Suntech, Medical Inc, Morrisville, NC, USA), rating of perceived exertion (Borg, 1970), respiratory exchange ratio (RER), and relative VO_2_ were recorded every 15s. The VO_2peak_ was determined when at least two of the following physiological criteria were met: a) RER > 1.10, b) A failure of heart rate to increase with an increase in workload and/or c) RPE >17 at the final stage. VO_2peak_ was considered the mean of the two highest 15-second values achieved during testing.

### Experimental trial

Two experimental trials followed a repeated-measures, randomized crossover design. All exercise trials were conducted in a climate-controlled environmental chamber (University of Arkansas, Fayetteville, AR, USA). Photographs illustrating the climate-controlled environmental chamber, exercise equipment, and placement of the Kestrel 4400 are provided in **Supplementary Figure 1**. Participants completed matched exercise in two separate environmental conditions with target setpoints of HD (39.2 °C and 30% RH) and WH (34.2 °C and 55% RH), both designed to achieve an equivalent WBGT of approximately 30 °C. Environmental conditions were established and stabilized before participants entered the chamber and were maintained throughout the exercise protocol. Trials were separated by at least six days and occurred at the same time of day to account for circadian rhythm. On arrival, participants provided a 24-hour urine sample to ensure euhydration, defined as urine specific gravity ≤ 1.020 (USG, Master-SUR, Atago Co Ltd, Tokyo, Japan) (Armstrong, 2007). One of our participants’ USG was >1.020, so we provided 500 mL of bottled water before the trial commenced. Nude body mass was assessed prior to trial instrumentation. Body mass with instrumentation was obtained when the participant mounted the cycle ergometer on a scale (VS-2501, WeighSouth.Inc, Asheville, NC, USA) before starting experimental trials. Participants then transitioned to the environmental chamber for a10 minutes seated baseline period. Participants then began a warm up for 2-5 minutes at a self-selected load, matched between trials. Participants then completed 60 minutes of cycling on a cycle ergometer (RacerMate, Seattle, WA, USA) at an individualized workload corresponding to 55% of the peak power output achieved during the graded VO_2peak_ test. The same individualized absolute workload was maintained during the HD and WH trials, and power output and cycling cadence were monitored throughout each trial to ensure adherence to the prescribed external workload. During 60 minutes of cycling, rectal temperature (T_re_), mean skin temperature (T_sk_), and body mass with instrumentation were measured every 10 minutes. Blood pressure, heart rate, forearm skin blood flux (FSBF), and local skin temperature were measured every 12 minutes. WBGT, ambient temperature, RH, and wind speed were measured every 15 minutes. Watts and cadence were measured by VeloTron CS 2008 (Racer Mate, Seattle, WA). Participants were provided water (warmed to 39 °C) every 15 minutes to replace approximately 50% of estimated body mass losses. Partial fluid replacement was used to attenuate dehydration while avoiding complete fluid replacement, thereby minimizing the confounding effects of excessive dehydration or overhydration (McDermott et al., 2017).

**Figure 1:**
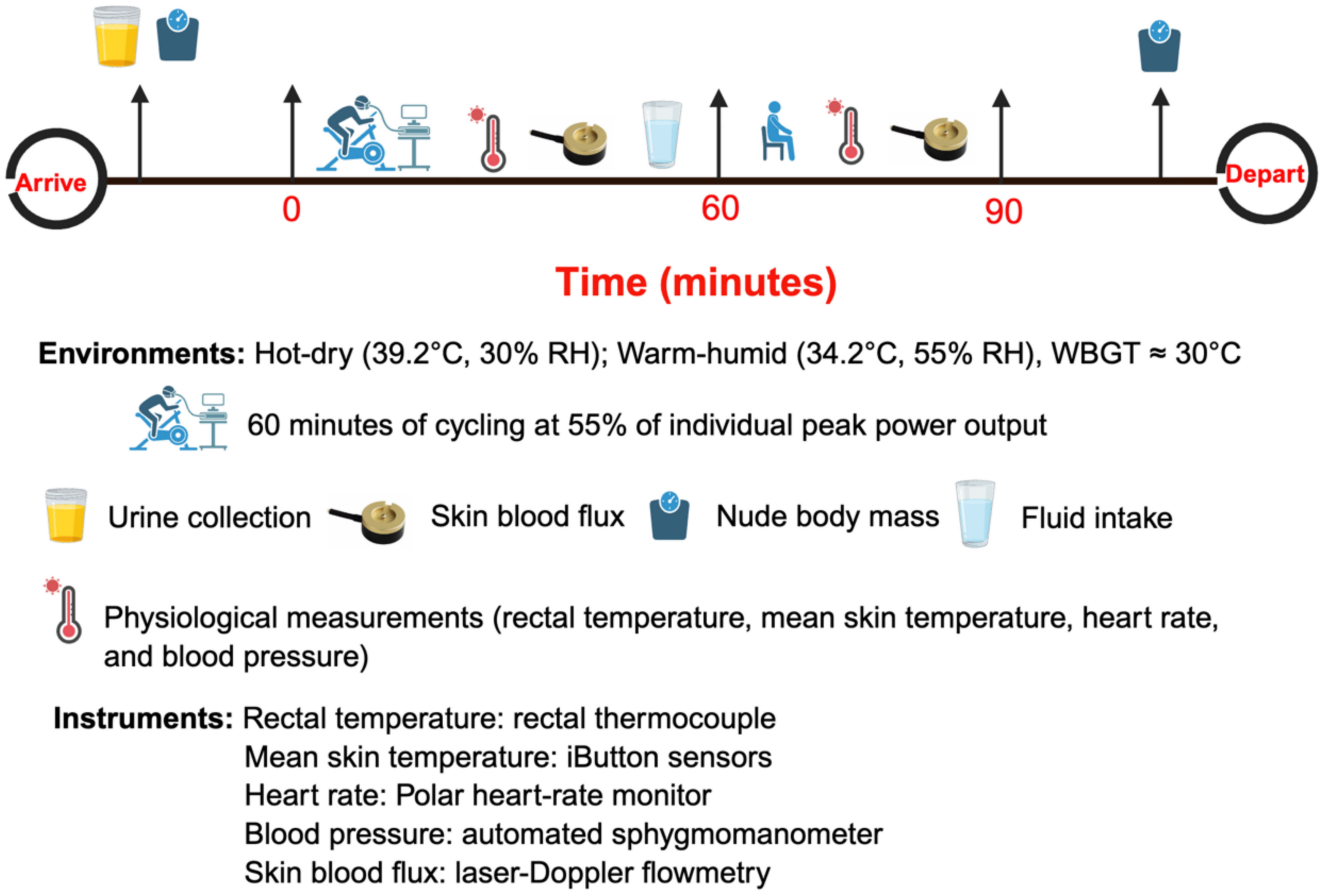
Schematic overview of the experimental protocol. Participants completed randomized crossover trials under hot-dry [HD; 39.2 °C, 30% relative humidity (RH)] and warm-humid (WH; 34.2 °C, 55% RH) conditions with equivalent wet-bulb globe temperature (WBGT; approximately 30 °C). Following pre-trial urine collection, nude body mass assessment, and instrumentation, participants completed 60 minutes of cycling at 55% of individual peak power output. Physiological measurements included rectal temperature, mean skin temperature, heart rate, blood pressure, and skin blood flux. Fluid was provided during exercise according to the study protocol. Following exercise, participants completed a 30-minute seated recovery period, maximal skin blood flux assessment, and post-trial nude body mass measurement. Source: BioRender (https://www.biorender.com).

After the 60 minutes of cycling, participants completed a 5 minutes cooldown on the cycle ergometer at a self-selected cadence and workload. Following the cooldown, participants got off the cycle ergometer and sat in a chair. Maximum skin blood flux was determined by heating forearm skin to 42°C for 30 minutes during recovery (Johnson et al., 1986; Pérgola et al., 1993; Kellogg et al., 1999). During a 30 minutes recovery period, participants were seated to have maximum skin blood flux measured via laser Doppler probe heating. After this, researchers removed instrumentation and measured nude body mass. Participants were provided refreshments, and future trials were scheduled, if necessary. A schematic overview of the experimental protocol, including the timeline, environmental conditions, exercise workload, physiological measurements, and principal instruments, is presented in **Figure 1**.

### Instrumentation

Total body heat loss and heat gain parameters were calculated using partitional calorimetry as described by Cramer and Jay (2019). Briefly, heat storage was calculated as metabolic heat production minus DHL, respiratory heat loss, and EHL. DHL was calculated as the sum of convective and radiative heat exchange, whereas conductive heat exchange was considered negligible. EHL was estimated from whole-body sweat rate, latent heat of vaporization, and sweating efficiency. Full equations (Equations 1–23), assumptions, and constants used for these calculations are provided in the **Appendix**. T_re_ was determined by a rectal thermocouple (RET-1, Physitemp Instrumental Inc, Clifton, NJ, USA) inserted ~15 cm past the anal sphincter before participants started experimental trials. T_sk_ was continuously measured using wireless temperature sensors (iButton, Maxim Integrated, San Jose, CA, USA) attached with porous zinc oxide tape to the chest, triceps, thigh, and calf on the right side of the body. Four-site weighted mean skin temperature was calculated using the Ramanathan (1964) equation: T_sk_ = 0.3 (T_chest_ + T_triceps_) + 0.2 (T_thigh_ + T_calf_). This four-site method was selected because it is commonly used in exercise and environmental physiology research and provides a practical estimate of whole-body mean skin temperature while minimizing participant instrumentation burden during cycling exercise. Although forehead and neck sites may contribute meaningfully to regional heat exchange, these sites are not included in the original Ramanathan four-site equation; therefore, the current site selection was used to remain consistent with the established equation and facilitate comparison with prior studies. BP and heart rate were measured via an automated blood pressure cuff (Tango II, Suntech, Medical Inc, Morrisville, NC, USA) with a 3-lead ECG and microphone. Red blood cell flux was assessed by laser Doppler flowmetry on the left dorsal forearm with a probe (laser-Doppler perfusion monitor and Probe 2b, Moor Instruments, Wilmington, DE, USA) held in place by a local heater (Periflux System 5000, Perimed, Ardmore, PA, USA) (Nilsson et al., 1980). T_re_, T_sk_, FSBF, and local skin temperature data were continuously recorded and stored using computer software for further analysis (LabChart 7; AD Instruments, Colorado Springs, CO, USA). Whole body sweat rate (WBSR) was calculated using the equation: WBSR = [(pre-body mass − post-body mass) − urine produced + fluid ingested]/exercise duration (McDermott et al., 2017). Ambient temperature, RH, WBGT, and wind speed were measured every 15 minutes throughout each experimental trial using a portable weather monitor (Kestrel 4400, Nielsen-Kellerman, Boothwyn, PA, USA). According to the manufacturer, the Kestrel 4400 has an accuracy of ±1.0 °C for ambient temperature, ± 3% for relative humidity, and ±3% of reading for wind speed measurements. The instrument was positioned adjacent to the cycle ergometer at approximately torso height and away from direct obstruction by the participant to verify the stability of environmental chamber conditions. Environmental conditions remained stable throughout the trials, as evidenced by the minimal variability in ambient temperature and relative humidity measurements presented in **Table 2**. The measurement ranges and manufacturer-reported accuracies of the principal instruments used during the experimental trials are summarized in **Supplementary Table 1**.

**Table 2:** Measured environmental conditions used to verify chamber stability, including ambient temperature, relative humidity (RH), wet-bulb globe temperature (WBGT), and wind speed, as well as fluid intake, whole-body sweat rate (WBSR), and urine specific gravity (USG) during the experimental trials [n = 10, mean (SD)].

Variable	HD	WH
Temperature (°C)	39.07 (0.21)	34.46 (0.81) **
RH (%)	33.25 (0.81)	58.79 (2.19) **
WBGT	30.1 (0.22)	30.0 (0.27)
Wind Speed (m/s) Fluid Intake (L)	2.44 (0.01) 0.82 (0.56)	2.42 (0.05) 0.65 (0.37)
WBSR (L/h)	1.68 (0.60)	1.39 (0.36)
USG	1.011 (0.005)	1.012 (0.004)

^*^ p *<* 0.05 between experimental conditions, ^**^p *<* 0.01 between experimental conditions.

### Statistical analysis

Data were analyzed with GraphPad Prism software (v.10.6.1). Descriptive statistics were calculated for all variables and are presented as mean ± standard deviation (SD). Linear mixed-effects models were used to assess differences in biophysical, thermal, and cardiovascular responses between HD and WH conditions over time, with condition and time treated as fixed effects and participant included as a random effect. Normality of residuals was assessed using the Shapiro–Wilk test. For variables that violated normality assumptions, non-parametric analyses were performed using the Friedman test to evaluate changes over time within each condition. When significant main effects were observed, *post hoc* pairwise comparisons were conducted using the Wilcoxon signed-rank test with Bonferroni adjustment. For parametric analyses, *post hoc* pairwise comparisons were performed with Bonferroni-adjusted alpha levels to identify significant differences between time points and conditions. Binary variables were analyzed using McNemar’s test. Statistical significance was set *a priori* at p ≤ 0.05.

## Results

**Table 2** presents the measured environmental conditions used to verify chamber stability, as well as WBSR, fluid intake, and USG for each experimental condition. As intended, ambient temperature (p < 0.001) and relative humidity (p < 0.001) differed significantly between HD and WH conditions. However, no differences were observed in WBGT (p = 0.566) or wind speed (p = 0.832) between conditions. Both HD and WH conditions were classified as moderate heat stress (Category 3; orange) based on WBGT criteria (Grundstein et al., 2015). No significant differences were observed between conditions for WBSR (p = 0.176), fluid intake (p = 0.127), or USG (p = 0.756).

Biophysical, thermal, and cardiovascular responses for the HD and WH conditions, together with the corresponding condition-effect statistical results and effect size are summarized in **Table 3**.

**Table 3:** Comparison of biophysical, thermal, and cardiovascular responses between hot-dry (HD) and warm-humid (WH) conditions.

Variable	n	HD, Mean (SD)	WH, Mean (SD)	Condition effect (p*)*	Effect size
DHL (W)	10	−80 (25)	48 (25)	< 0.001	ηp² = 0.950
EHL (W)	10	450 (53)	319 (30)	< 0.001	ηp² = 0.932
Heat storage (W)	10	285 (115)	245 (81)	0.323	ηp² = 0.347
Cumulative heat storage (kJ)	10	1058 (350)	977 (288)	0.364	*d* = 0.300
T_re_ (°C)	10	38.31 (0.79)	38.29 (0.71)	0.885	ηp² = 0.009
T_sk_ (°C)	8	36.28 (0.67)	35.89 (0.70)	0.030	ηp² = 0.465
Heart rate (bpm)	10	146 (40)	143 (40)	0.690	ηp² = 0.147
SBP (mmHg)	9	166 (39)	164 (38)	0.876	ηp² < 0.001
DBP (mmHg)	9	62 (17)	61 (16)	0.737	ηp² = 0.143
MAP (mmHg)	9	93 (17)	96 (13)	0.926	ηp² = 0.121
FSBF (AU)	10	113 (59)	95 (44)	0.324	ηp² = 0.001
%CVCmax (AU/mmHg)	10	0.65 (0.35)	0.61 (0.27)	0.646	ηp² < 0.001

Hot-dry; WH, warm-humid; DHL, dry heat loss; EHL, evaporative heat loss; T_re_, rectal temperature; T_sk_, mean skin temperature; SBP, systolic blood pressure; DBP, diastolic blood pressure; MAP, mean arterial pressure; FSBF, forearm skin blood flux; CVC_max_, maximal cutaneous vascular conductance; AU, arbitrary units; ηp², partial eta squared; d, Cohen’s d. Effect sizes are reported as partial eta squared (ηp²) for outcomes analyzed using linear mixed-effects models and Cohen’s d for cumulative heat storage. Statistical significance was set at p ≤ 0.05.

### Biophysical responses

**Figure 2:**
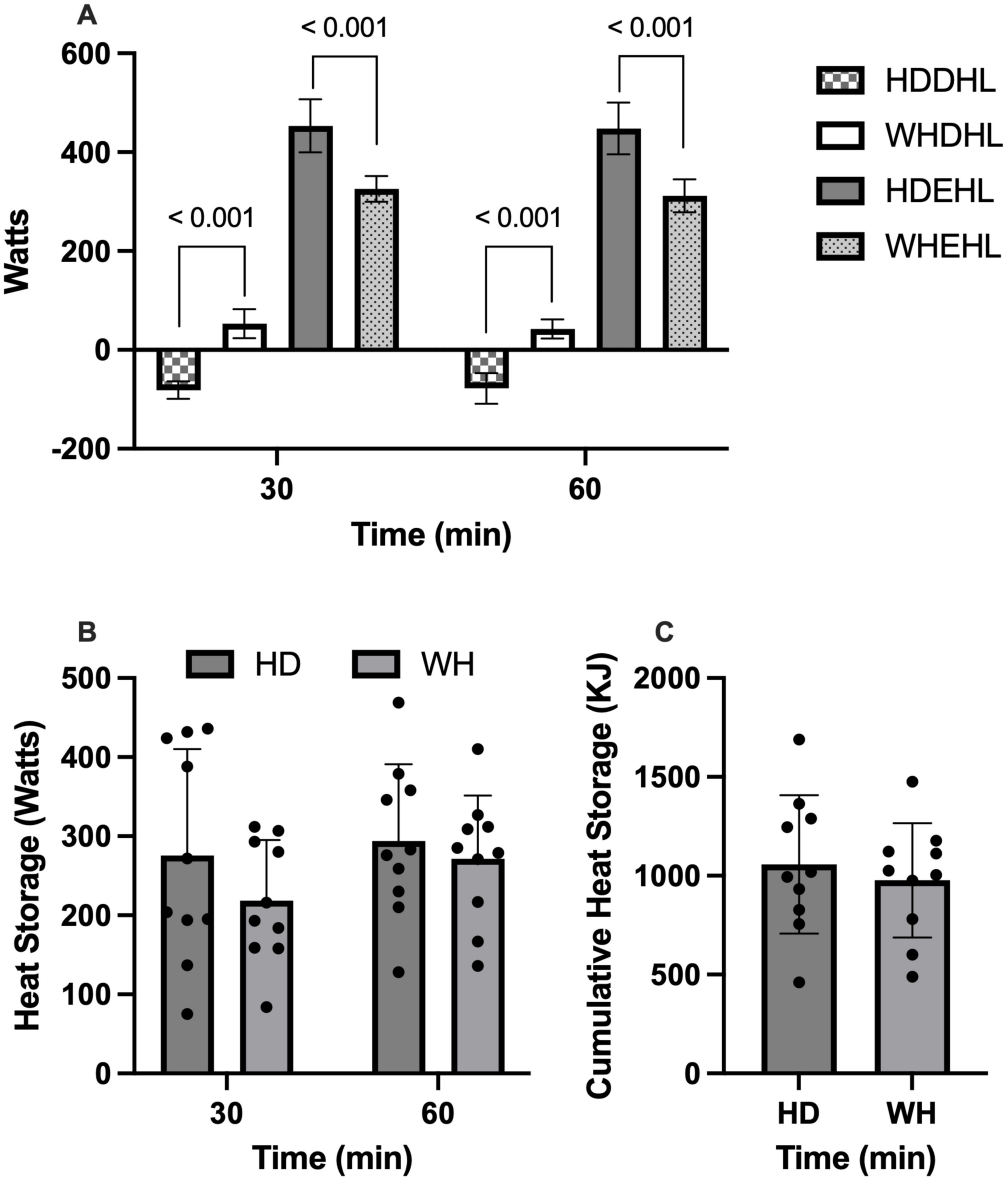
Changes in dry heat loss (DHL), evaporative heat loss (EHL), heat storage, and cumulative heat storage during 60 minutes of cycling between hot-dry (HD) and warm-humid (WH) environmental conditions. DHL and EHL [**(A)**, n = 10], heat storage [**(B)**, n = 10], cumulative heat storage [**(C)**, n =10]. Data are presented as mean (SD).

DHL was significantly greater in WH than HD (p < 0.001), with no main effect of time (p = 0.438) or condition × time interaction (p = 0.130). DHL remained negative throughout the exercise protocol in HD, indicating net dry heat gain, whereas positive values in WH indicated net dry heat loss (**Figure 2A**; **Table 3**). Conversely, EHL was significantly greater in HD than WH (p < 0.001), with no main effect of time (p = 0.170) or condition × time interaction (p = 0.523). Heat storage did not differ over time (p = 0.116), between conditions (p = 0.323), or for the condition × time interaction (p = 0.430; **Figure 2B**; **Table 3**). Similarly, cumulative heat storage did not differ between conditions (p = 0.364; **Figure 2C**; **Table 3**).

### Thermal responses

**Figure 3:**
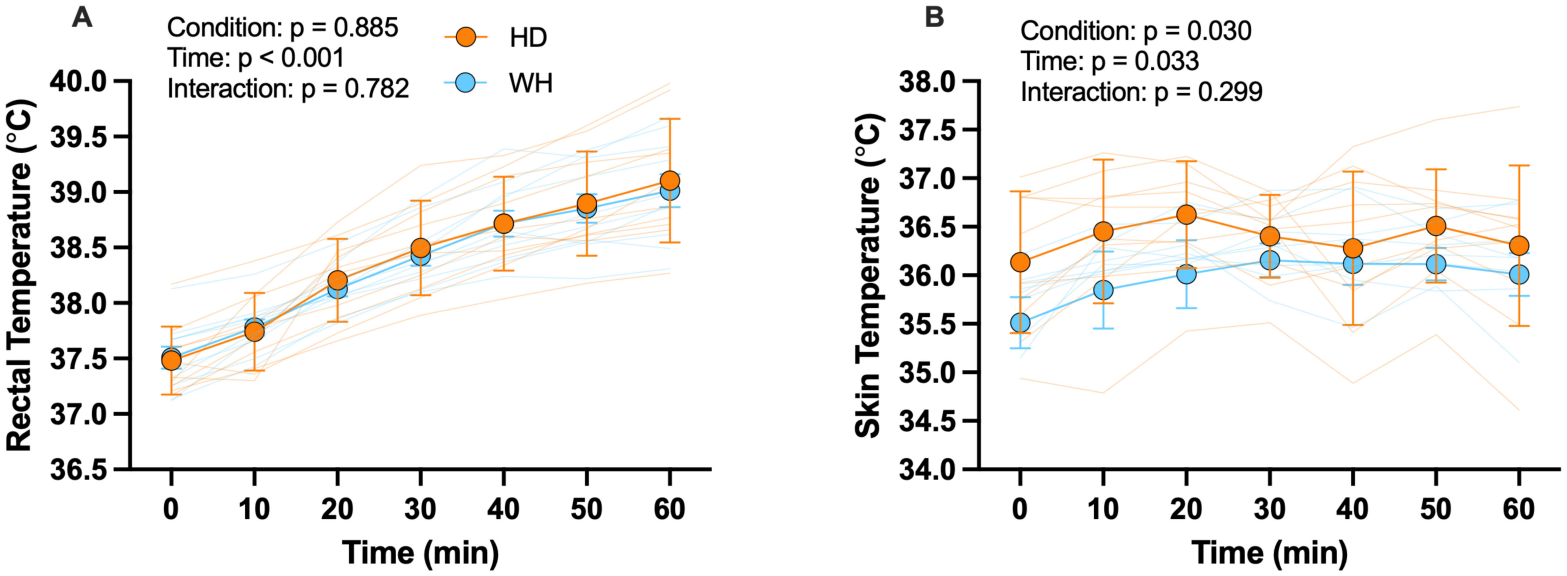
Thermal responses during 60 minutes of cycling between hot-dry (HD) and warm-humid (WH) environmental conditions. Rectal temperature [**(A)**, n = 10], skin temperature [**(B)**, n = 8]. Data are presented as mean (SD).

T_sk_ data were unavailable for two participants because the iButton sensors detached during exercise or the recorded temperature data could not be retrieved. Therefore, analyses involving T_sk_ included eight participants. T_re_ increased significantly over time (p < 0.001), with no main effect of condition (p = 0.885) or condition × time interaction (p = 0.782; **Figure 3A**; **Table 3**). T_sk_ demonstrated significant main effects of time (p = 0.033) and condition (p = 0.030), with greater values in HD than WH, but no condition × time interaction (p = 0.299; **Figure 3B**; **Table 3**).

### Cardiovascular responses

**Figure 4:**
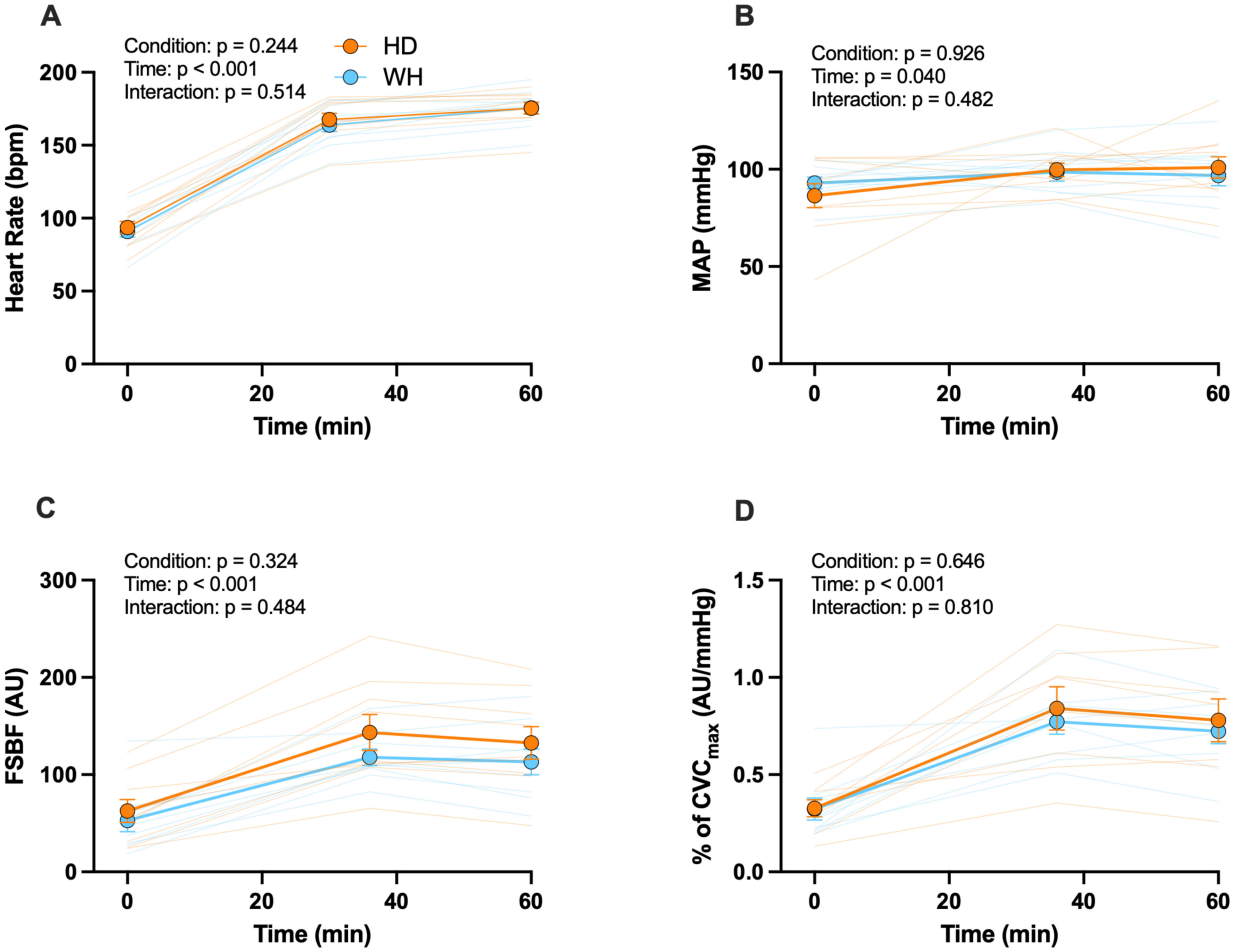
Cardiovascular responses during 60 minutes of cycling between hot-dry (HD) and warm-humid (WH) environmental conditions. Heart rate [**(A)**, n = 10], mean arterial pressure [MAP, **(B)**, n = 9], forearm skin blood flux [FSBF, **(C)**, n =10], percentage of maximal cutaneous vascular conductance [% of CVC_max_, **(D)**, n =10]. Data are presented as mean (SD).

Heart rate increased significantly over time (p < 0.001), with no main effect of condition (p = 0.244) or condition × time interaction (p = 0.514; **Figure 4A**; **Table 3**). SBP and DBP also increased over time (both p < 0.001), whereas MAP demonstrated a significant time effect (p = 0.040). No condition effects or condition × time interactions were observed for SBP, DBP, or MAP (all p > 0.05; **Figure 4B**; **Table 3**). FSBF and %CVC_max_ increased significantly over time (both p < 0.001), with no condition effects or condition × time interactions (all p > 0.05; **Figures 4C, D**; **Table 3**).

## Discussion

The current study was designed to compare heat storage and physiological responses to different environmental conditions (HD versus WH) but similar WBGT during moderate intensity cycling. Our primary findings support similar thermoregulation responses to previous literature based on minimal or light exercise. With moderate intensity exercise, our data indicate no differences in heat storage, T_re_, heart rate, SBP, DBP, MAP, FSBF, and %CVC_max_ between HD and WH conditions. However, EHL and T_sk_ in HD were significantly greater than in WH, while DHL in WH was significantly greater than HD. Hence, the results of the present study demonstrate that moderate heat stress of varying conditions, but similar WBGT, stimulated matched cardiovascular responses, yet altered thermoregulation to accommodate moderate intensity 60 minutes exercise in the heat. Likely, convective heat losses were increased during WH conditions to allow thermoregulatory similarities, despite our uncompensable heat stress. Our findings suggest that WBGT alone may not fully characterize the physiological mechanisms of heat exchange across different environmental conditions, particularly during moderate-intensity exercise. Occupational, military, or athletic groups should be cautious when following any guidelines, recommendations, standards, or protocols for heat stress based on the WBGT assessment, if work rate is not taken into consideration for activity modifications.

The primary mechanism of heat dissipation in the current study can be explained by differences in DHL and EHL between HD and WH conditions. DHL in HD was negative, which revealed heat gain compared to our WH condition. In the present study of both HD and WH environmental conditions, heat balance was mainly determined by convection and evaporation. Under our HD condition, the ambient temperature was greater than T_sk_ (**Table 2**), and heat transferred from the environment to the body. In addition, fan use increases ambient air movement, which further increases heat gain via convection when there is a gradient from the environment to the skin (Cramer and Jay, 2019). According to previous research on ambient temperature thresholds where fan use is beneficial, using a fan is an effective strategy for cooling the human body when air temperature is < 39°C (Morris et al., 2021). In contrast, convective heat loss is enhanced when the air temperature is lower than T_sk_; as seen in our WH condition. In addition, fan use in our WH condition (34.5°C) boosts convection due to ambient temperature < 39°C. Regarding EHL in our WH condition, high relative humidity decreases evaporative efficacy (Alber-Wallerström and Holmér, 1985). A recent study also demonstrated that sweat can be fully evaporated at low relative humidity (< 35%), but evaporation of sweat is restricted when relative humidity is > 55% (Beigtan et al., 2024). Under our WH condition, EHL was limited due to high relative humidity (58.79%). In contrast, sweat can be fully evaporated in our HD condition because of lower relative humidity (33.25%). In conclusion, our data suggest that heat balance in HD is limited by convection rather than evaporation of sweat, whereas heat balance in WH was limited by the evaporation of sweat.

No difference in heat storage and cumulative heat storage between HD and WH conditions indicated that heat loss from evaporation in HD was partly offset by the heat gain from convection and radiation, consistent with previous studies with WBGT 27.8–30°C (Stapleton et al., 2012; Foster et al., 2022). However, Keatisuwan et al. (1996) found heat storage in HD (ambient temperature: 40°C, RH: 30%, WBGT: 32°C) was greater compared to WH (ambient temperature: 31°C, RH: 80%, WBGT: 32°C) conditions during 60 minutes of cycling at 40% V O_2peak_. The divergent results from previous studies indicated that there may be a threshold near 32°C WBGT before the difference in heat storage is identified.

Consistent with our heat storage findings, an equivalent WBGT ~30°C resulted in a similar T_re_ between HD and WH conditions during 60 minutes of moderate-intensity cycling. Previous studies agree with our findings with matched WBGT (27.8–30°C) of combining varying ambient temperature (20–46°C) and relative humidity (10–84%) (Smolander et al., 1987; Stapleton et al., 2012; Seo et al., 2019; Bartman et al., 2024). For example, one of the studies used similar WBGT (30°C) and exercise type (cycling) to the present investigation (Seo et al., 2019). Previous data suggest T_re_ was no different between HD and WH conditions during 60 minutes of cycling at a fixed work rate of 350 watts. In contrast, Keatisuwan et al. (1996) reported that core temperature in HD (ambient temperature: 40°C, RH: 30%) was significantly greater than WH (ambient temperature: 31°C, RH: 80%) at equivalent WBGT during 60 minutes of cycling at 40% V O_2peak_. More extreme heat stress in that study may explain the differences in core temperature between HD and WH conditions (Keatisuwan et al., 1996). Future research should investigate whether there is a threshold of WBGT before differences may be observed between HD and WH conditions.

T_sk_ reflects temperature changes in the peripheral shell of the human body. In the current study, T_sk_ was greater in HD than WH, consistent with previous studies comparing HD and WH conditions with equivalent WBGT values ranging from 27.8 to 32 °C (Smolander et al., 1987; Keatisuwan et al., 1996; Seo et al., 2019; Bartman et al., 2024). The greater T_sk_ observed in HD was likely attributable, in part, to the higher ambient temperature in HD (~39 °C) than WH (~34.5 °C). In addition, greater dry heat gain occurred in HD than WH. This transfer of dry heat from the environment to the skin may have contributed to the higher T_sk_ observed in HD. Greater interindividual variability in T_sk_ was also evident in the HD condition, including at the initial measurement, and remained apparent throughout exercise. Because this variability was present at the beginning of the trial, it may not be attributable solely to subsequent exercise-induced thermal responses and may partly reflect individual differences in regional skin temperature or local factors associated with skin-temperature measurement. Furthermore, T_sk_ analyses included eight participants because data from two participants were unavailable due to iButton sensor detachment or unreadable recordings. Therefore, the condition effect for T_sk_ should be interpreted cautiously given the small sample size and observed interindividual variability.

Integrative cardiovascular responses to heat stress during prolonged exercise depends on thermal stress and exercise intensity (Chou and Coyle, 2023). In the current study, we found no cardiovascular differences between conditions. Heart rate is widely used to assess physiological strain in combination with core temperature during exercise in the heat (Stapleton et al., 2012). During exercise in the heat, more blood is distributed to the skin for heat dissipation. Heart rate increases to maintain cardiac output and meet the demands for maintaining blood pressure, heat dissipation, and exercising muscle perfusion (Chou and Coyle, 2023). Our data confirmed that heart rate increased throughout 60 minutes of cycling in both HD and WH conditions. In contrast, there was no difference in HR between HD and WH, which is consistent with findings from previous studies (Hayes et al., 2014; Seo et al., 2019; Bartman et al., 2024). Heart rate in the current study reached over 86% age-predicted maximum heart rate after 30 minutes of cycling (**Figure 3**), which corresponds to vigorous intensity exercise (Feito and Magal, 2022). The heart rate data demonstrated that the exercise intensity ranged from moderate to vigorous during our trials. In the current study, SBP and MAP increased and reached a maximum around 30 minutes of cycling and were maintained throughout the protocol. In contrast, DBP was maintained during the 60 minutes of cycling.

Skin blood flow increases substantially in response to thermal stress via vasodilation (Charkoudian, 2003). In the present study, FSBF increased throughout the 60 minutes of cycling. Although T_sk_ in HD condition was higher than WH, FSBF was no different between HD and WH conditions. The amplitude of change in the skin blood flux during exercise in the heat is mediated by the core-to-skin temperature gradient (Johnson and Park, 1979; Pérgola et al., 1996). The rate of change in skin blood flux is a priority driven by central (i.e., visceral and brain) thermoreceptors at any temperature gradient compared to peripheral (i.e., skin) thermoreceptors (Périard et al., 2021). Our data revealed that T_re_ was not significantly different between HD and WH conditions, which likely accounted for similar responses in skin blood flux between HD and WH conditions, despite T_sk_ differences. Altogether, under exercise-heat stress, the cardiovascular system dynamically adjusted and balanced the competition between skin and muscle for large fractions of cardiac output (Rowell, 1993).

Examining the effectiveness of heat stress indices in differentiating the severity of environmental heat stress is important for developing guidelines and strategies to mitigate heat-related health consequences. Both HD and WH conditions in the present study were classified as moderate heat stress (Category 3; orange) according to WBGT criteria (Grundstein et al., 2015), for which recommendations permit up to 120 minutes of continuous activity under these environmental conditions, depending on work intensity and other modifying factors. However, these recommendations may not fully account for differences in metabolic demands and environmental characteristics. Although substantial increases in rectal temperature occurred during the 60 minutes exercise protocol, the present data do not permit accurate prediction of physiological responses beyond the experimental duration. Therefore, caution should be exercised when extrapolating these findings to longer periods of exercise or work in the heat. Nevertheless, our findings suggest that both environmental characteristics and metabolic demands should be considered when applying WBGT-based heat stress recommendations during moderate-intensity exercise.

Our findings should be taken with caution based on limitations. First, it would be rare to continue steady-state physical activity or work in living situations when people experience prolonged heat stress. Our experimental design did not allow for heat stress mitigating strategies, such as reducing workload, rest breaks or slowing down. Second, the present study was limited to 60 minutes of exercise and therefore cannot characterize the longer-term trajectory of core temperature responses in either environmental condition. Future studies employing longer exercise durations are needed to determine whether thermoregulatory responses eventually diverge between hot-dry and warm-humid environments. Third, mean skin temperature was estimated using the Ramanathan four-site equation. Although this method is widely used, it may not fully capture regional skin temperature differences at sites not measured in the present study, such as the forehead or neck, which may contribute to heat exchange during exercise in the heat. Fourth, although the randomized crossover design reduced between-subject variability by allowing each participant to serve as his own control, the exclusion of five participants resulted in a final sample of ten participants, which may have reduced statistical power to detect smaller differences in secondary outcomes and limits the generalizability of the findings. Furthermore, an *a priori* power analysis was not performed, and therefore the possibility of a type II error for some outcomes cannot be excluded. Fifth, environmental conditions were monitored using a portable weather monitor rather than a fixed laboratory-grade environmental monitoring system. Although the Kestrel 4400 may provide lower precision than laboratory-grade instruments, the small variability in measured environmental conditions suggests that this limitation likely had little influence on the study findings. Finally, our data were collected in young, physically fit men in whom thermoregulatory capacity is likely preserved. The relatively homogeneous aerobic fitness of the participants (all VO_2peak_ ≥ 45 mL/kg/min), combined with the small sample size, limited the suitability of exploratory analyses examining whether VO_2peak_ or body fat percentage influenced the observed responses. Future studies with larger samples and broader ranges of aerobic fitness and body composition are needed to determine whether these characteristics modify responses under HD and WH conditions. Consequently, these findings may not be generalizable to women, older adults, children, or other vulnerable populations. Females may demonstrate different thermoregulatory responses due to differences in body composition, sweating characteristics, skin blood flow, and hormonal fluctuations throughout the menstrual cycle. Future studies should investigate whether the present findings are consistent across these populations.

## Conclusion

The findings of our study provide important insight regarding heat storage, thermoregulatory and cardiovascular responses to heat stress of varying environmental conditions with equivalent WBGT during moderate-intensity exercise. Our data reveal hot environments of equivalent WBGT alter thermoregulation to accommodate for differences in humidity, which confirmed previous findings and extend data to include more extreme environments and greater exercise intensity. It is noteworthy that our data support WBGT as a reliable heat stress indicator for assessing thermal and cardiovascular strain in varying environmental conditions during moderate intensity exercise. However, these findings suggest that WBGT alone may not fully capture physiological strain under all combinations of environmental conditions and metabolic demands, and caution should be taken when occupational, military, or athletic groups use guidelines, recommendations, standards, or protocols for heat stress based on the WBGT assessment, unless work rate is considered. Future investigations should examine similar heat stress in the aged, women, children, or other vulnerable populations.

## Data Availability

The raw data supporting the conclusions of this article will be made available by the authors, without undue reservation.

## Appendix

### Metabolic heat production calculations

Metabolic heat production (H_prod_) was estimated with the equation (Cramer and Jay, 2019):

(1)
$${\text{H}}_{\text{prod}}=\text{M}–{\text{W}}_{\text{k}}(\text{W})$$

where M is metabolic energy expenditure and W_k_ is external work rate. M was calculated using the equation (Consolazio et al., 1963):

(2)
$$\text{M}={\text{VO}}_{2}\times (((((\text{RER}–0.7)/0.3)\times 21.3)+(((1.0–\text{RER})/0.3)\times 19.62))/60)\times 1,000(\text{W})$$

VO_2_ and CO_2_ were measured from expired gas for each subject (see above for procedure), and RER was calculated using VO_2_ and VCO_2_:

(3)
$$\text{RER}={\text{VCO}}_{2}/{\text{VO}}_{2}$$

Wk was calculated using the 55% of wattage reached during the last stage of the V^•^ O_2peak_ test.

### Rate of heat storage calculations

Heat storage (S) was calculated at 30 and 60 min throughout the protocol, calculated as:

(4)
$$\text{S}={\text{H}}_{\text{prod}}–{\text{H}}_{\text{dry}}–{\text{H}}_{\text{res}}–\text{EHL}(\text{W})$$

where H_dry_ represents the sum of dry heat losses from radiation (R) and convection (C), H_res_ represents heat loss from respiration, and EHL represents heat loss from evaporation. The rate of heat loss from conduction was considered negligible and thus was eliminated from the equation (Cramer and Jay, 2019).

Total H_dry_ was calculated as the sum of C+R where C Equation 5 and R Equation 6 were calculated as:

(5)
$$\text{C}={\text{h}}_{\text{c}}\times ({\text{T}}_{\text{sk}}–{\text{T}}_{\text{o}})*\text{BSA}(\text{W})$$

(6)
$$\text{R}={\text{h}}_{\text{r}}\times ({\text{T}}_{\text{sk}}–{\text{T}}_{0})*\text{BSA}(\text{W})$$

where T_o_ is the operational temperature, T_sk_ is mean skin temperature, and h_c_ and h_r_ are the convective heat transfer coefficient and radiative heat transfer coefficient, respectively (Vargas et al., 2019). Since radiant heat was considered negligible, ambient temperature (T_a_) was used instead of operational temperature (T_o_): T_o_=T_a_.

H_c_ value for cycle ergometry was calculated using the equation (Lotens and Havenith, 1991):

(7)
$${\text{H}}_{\text{c}}=8.3\times {\left(0.07+0.0043\times {\text{f}}_{\text{ped}}+{\text{V}}_{\text{air}}\right)}^{0.86}$$

Where f_ped_ is pedalling frequency (revolutions/min), V_air_ is air velocity (m/s).

H_r_ was calculated as (Cramer and Jay, 2019):

(8)
$${\text{H}}_{\text{r}}=4\text{ϵσ}\frac{A_{r}}{A_{D}}[273.2+\frac{t_{sk}+t_{r}}{2}](\text{W}/{\text{m}}^{2}/\text{K})$$

where ϵ is the nondimensional emissivity of the body surface, assumed 0.95 for clothed skin, σ is the Stefan-Boltzmann constant (5.67 × 10^−8^ W/m^2^/K^4^), A_r_/A_D_ was assumed 0.70 for a sitting individual (Winslow et al., 1936; Tanabe et al., 2000).

BSA was calculated as:

(9)
$$\text{BSA}=0.007184\times ({\text{height}}^{0.725})\times ({\text{weight}}^{0.425})$$

Whole body sweat rate (WBSR) was calculated as:

(10)
$$\text{WBSR}=[({\text{M}}_{\text{pre}}-{\text{M}}_{\text{post}})-\text{urine produced}+\text{fluid ingested}]/60$$

where M_pre_ is the initial mass determination, M_post_ is the final mass determination, and 60 is the total minutes of the protocol.

H_res_ was calculated using M as:

(11)
$${\text{H}}_{\text{res}}={\text{C}}_{\text{res}}+{\text{E}}_{\text{res}}(\text{W})$$

(12)
$${\text{C}}_{\text{res}}=[0.0014\times (\text{M}–{\text{W}}_{\text{k}})\times (34–{\text{T}}_{\text{a}})](\text{W})$$

(13)
$${\text{E}}_{\text{res}}=[0.0173\times (\text{M}–{\text{W}}_{\text{k}})\times (5.87–{\text{P}}_{\text{a}})](\text{W})$$

where P_a_ is the partial pressure of water vapor in ambient air as kPa calculated as (Vargas et al., 2019):

(14)
$$\text{Pa}={\text{P}}_{\text{a},\text{s}}\times (\text{RH}/100)$$

where P_a,s_ is the saturated water vapor pressure of the air:

(15)
$${\text{P}}_{\text{a},\text{s}}=(\text{EXP}(18.956–(4030.18/({\text{T}}_{\text{a}}+235))))/10$$

EHL was estimated using measurements of WBSR Equation (1) and sweating efficiency (S_eff_) with the equation:

(16)
$$\text{EHL}=\text{WBSR}\times \lambda \times {\text{S}}_{\text{eff}}/60(\text{W})$$

Where *λ* is the latent heat vaporization of sweat in (2,426 J/g), and 60 represents the conversion from minutes to seconds for calculating evaporative heat loss in watts.

Sweating efficiency was calculated as:

(17)
$${\text{S}}_{\text{eff}}=1–(\omega_{\text{req}}^{2}/2)(\text{ND})$$

Skin wettedness required to achieve heat balance (*ω*) was calculated as:

(18)
$$\omega_{\text{req}}={\text{E}}_{\text{req}}/{\text{E}}_{\text{max}}/\omega_{\text{max}}(\text{ND})$$

(19)
$${\text{E}}_{\text{req}}={\text{H}}_{\text{prod}}–{\text{H}}_{\text{dry}}–{\text{H}}_{\text{res}}(\text{W})$$

(20)
$${\text{E}}_{\text{max}}=({\text{P}}_{\text{sk},\text{s}}–{\text{P}}_{\text{a}})/({\text{R}}_{\text{e},\text{cl}}+(1/({\text{h}}_{\text{e}}\times {\text{f}}_{\text{cl}})))\times {\text{A}}_{\text{D}}(\text{W})$$

where *ω*_max_ was assumed 0.85 for an aerobically trained, unacclimated subject (Ravanelli et al., 2018). P_sk,s_ is water vapor pressure at the skin when saturated, R_e,cl_ is the evaporative heat transfer resistance of clothing ensemble assumed 0.005, similar to measured R_e,cl_ for an athletic ensemble of shorts, t shirt, socks, and sneakers (Mccullough et al., 1985), h_e_ is the evaporative heat transfer coefficient of air adjacent to the clothing layer, f_cl_ is the clothing area factor (1.031 ND), and A_D_ is body surface area calculated from the standard DuBois equation (DuBois and DuBois, 1916).

h_e_ was calculated as:

(21)
$${\text{h}}_{\text{e}}=16.5\times {\text{h}}_{\text{c}}\times (760/{\text{P}}_{\text{b}})\;(\text{W}/{\text{m}}^{2}/\text{a})$$

where P_b_ is barometric pressure in mmHg.

f_cl_ was calculated as:

(22)
$${\text{f}}_{\text{cl}}=1+(0.31\times 0.1)\;(\text{ND})$$

where 0.1 clo is the insulation factor (I_cl_) of subject attire (ISO, 2007).

Cumulative S was calculated as the sum of total heat storage in kJ determined by the S (in W) at each time-point (30 and 60 min):

(23)
CumulativeS=(S×s/1,000) (kJ)

Where s is the interval (in second) between the previous time-point. The summation of all two time-points was cumulative S.
