# Supplementary material for: Thermoregulation in hot-dry and warm-humid heat stress during cycling among males
Source: Front Physiol. 2026 Aug 5;17:1856143. doi: 10.3389/fphys.2026.1856143 (PMC13485807; doi:10.3389/fphys.2026.1856143)
Supplement: Supplementary file 1 [file DataSheet1.docx]

**Supplementary Table S1. Technical specifications of the principal measurement instruments used during the experimental trials**

| **Instrument/model** | **Manufacturer** | **Measured parameter** | **Measurement range** | **Manufacturer-reported accuracy** |
| --- | --- | --- | --- | --- |
| Kestrel 4400 Heat Stress Tracker | Nielsen-Kellerman, Boothwyn, PA, USA | Ambient temperature | −29.0 to 70.0°C | ±1.0°C |
| Kestrel 4400 Heat Stress Tracker | Nielsen-Kellerman, Boothwyn, PA, USA | Relative humidity | 5–95% RH | ±3% RH |
| Kestrel 4400 Heat Stress Tracker | Nielsen-Kellerman, Boothwyn, PA, USA | Wind speed | 0.6–40.0 m/s | ±3% |
| RET-1 rectal probe/thermocouple | Physitemp Instruments Inc., Clifton, NJ, USA | Rectal temperature | 0–90°C | ±0.1°C |
| DS1922L Thermochron iButton | Maxim Integrated/Analog Devices, San Jose, CA, USA | Skin temperature | −40 to 85°C | ±0.5°C from −10 to 65°C |
| Tango II stress test monitor | SunTech Medical Inc., Morrisville, NC, USA | Blood pressure | 30–270 mmHg | ±2% or ±3 mmHg |
| Tango II stress test monitor | SunTech Medical Inc., Morrisville, NC, USA | Heart rate | 40–200 beats/min | ±2% or ±1 beat/min |
| Laser-Doppler perfusion monitor and Probe 2b | Moor Instruments, Wilmington, DE, USA | Forearm skin blood flux | NR | NR |

**Note:** RH, relative humidity; NR, not reported or not applicable. Measurement ranges and accuracies are based on manufacturer-reported specifications. Skin blood flux is reported in arbitrary/perfusion units, and a conventional physiological measurement range and accuracy are not reported in the same manner as temperature, humidity, or blood pressure measurements.

**Supplementary Figure S1. Climate-controlled environmental chamber and experimental setup.** Photograph illustrating the environmental chamber layout, cycle ergometer, physiological measurement equipment, and placement of the Kestrel 4400 Heat Stress Tracker.
